# Supplementary material for: Correction of a widespread bias in pooled chemical genomics screens improves their interpretability
Source: Mol Syst Biol. 2024 Sep 30;20(11):3. doi: 10.1038/s44320-024-00069-y (PMC11535069; doi:10.1038/s44320-024-00069-y)
Supplement: Supplementary file 2 — Appendix [file 44320_2024_69_MOESM2_ESM.pdf]

Appendix

Correction of a widespread bias in pooled chemical genomics screens improves their interpretability

Lili M. Kim[1,*], Horia Todor[1,#,*], Carol A. Gross[1,2,3]

Affiliations:
[1]Department of Microbiology and Immunology, University of California, San Francisco, San Francisco, California, USA.
[2]Department of Cell and Tissue Biology, University of California, San Francisco, San Francisco, California, USA.
[3]California Institute of Quantitative Biology, University of California, San Francisco, San Francisco 94158, CA, USA.

*These authors contributed equally to this work.
[#]Correspondence: horia.todor@gmail.com

Table of Contents

Appendix Figure S1: Bin size does not drastically affect normalization. ………………………………………………………………………………………... 2

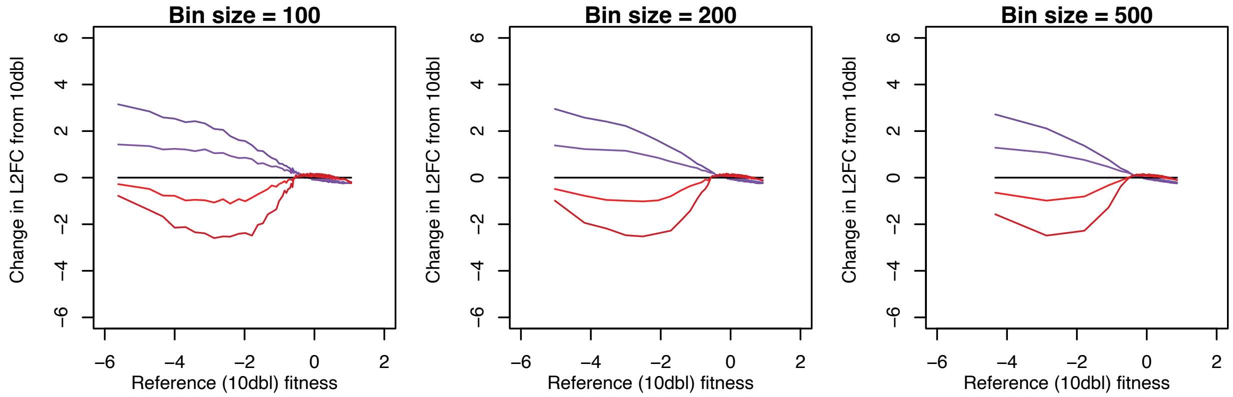

Appendix Figure S1. Bin size does not drastically affect normalization. Experimental time course data from (Rishi *et al*, 2020) normalized to a 10-doubling reference L2FC using a bin size of 100 (A, 0.3% library size), 200 (B, same as Figure 1C, 0.6% library size), and 500 (C, 1.5% library size). Bin size did not drastically affect the applied correction.
