# Supplementary material for: Correction of a widespread bias in pooled chemical genomics screens improves their interpretability
Source: Mol Syst Biol. 2024 Sep 30;20(11):3. doi: 10.1038/s44320-024-00069-y (PMC11535069; doi:10.1038/s44320-024-00069-y)
Supplement: Supplementary file 4 — Expanded View Figures [file 44320_2024_69_MOESM4_ESM.pdf]

Expanded View Figures

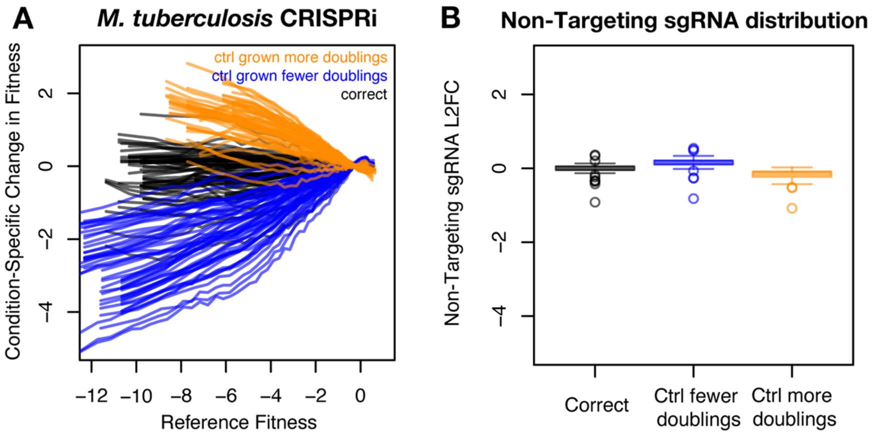

Figure EV1. Using deliberately incorrect controls induces correlations between the reference and condition-specific fitnesses in the *M. tuberculosis CRISPRi* data, but does not affect the non-targeting control sgRNAs.

(**A**) Differences in the number of library doublings between the experimental and control conditions results in positive or negative correlations between reference and condition-specific fitnesses. For all drugs, we compared each timepoint (1, 5, or 10 days pre depletion) to a control with fewer (blue lines), the same (black lines), or more (orange lines) cell doublings (Methods, Table EV1). The incorrect comparisons resulted in strong correlations between reference and condition-specific change in fitness. If the control was grown for more cell doublings (e.g., longer pre-depletion, orange lines), sick strains would appear protected (positive condition-specific change in fitness) since they would be less depleted in the treated sample. If the control was grown for fewer cell doublings (e.g., shorter pre-depletion, blue lines), sick strains would appear sensitized (negative condition-specific change in fitness) since they would be more depleted in the treated sample. (**B**) The condition-specific change in fitness of non-targeting sgRNAs is not strongly affected by using non-matched control samples. The median of the non-targeting controls: correct comparison = 0.00, control with fewer doublings = 0.16, control with more doublings = -0.16.

 © The Author(s)

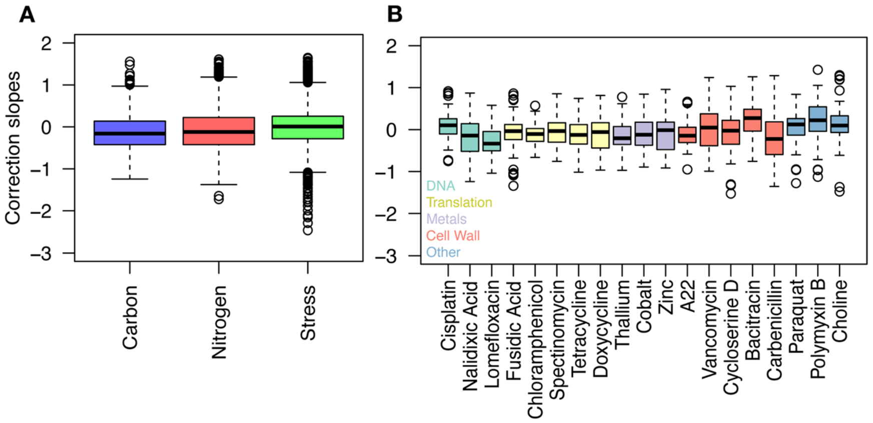

Figure EV2. Strong negative- or positive relationships between reference and condition-specific fitnesses are not drug-specific.

For each experiment in (Price et al, 2018), we calculated the slope of the bin median reference and condition-specific fitnesses. A slope of zero indicates a perfectly matched experiment. Neither broad categories of stresses (**A**) nor specific drugs (**B**) resulted in consistently low or high slopes, suggesting that nonzero relationships between reference and condition-specific fitnesses are due to experimental vagaries, not biological effects.

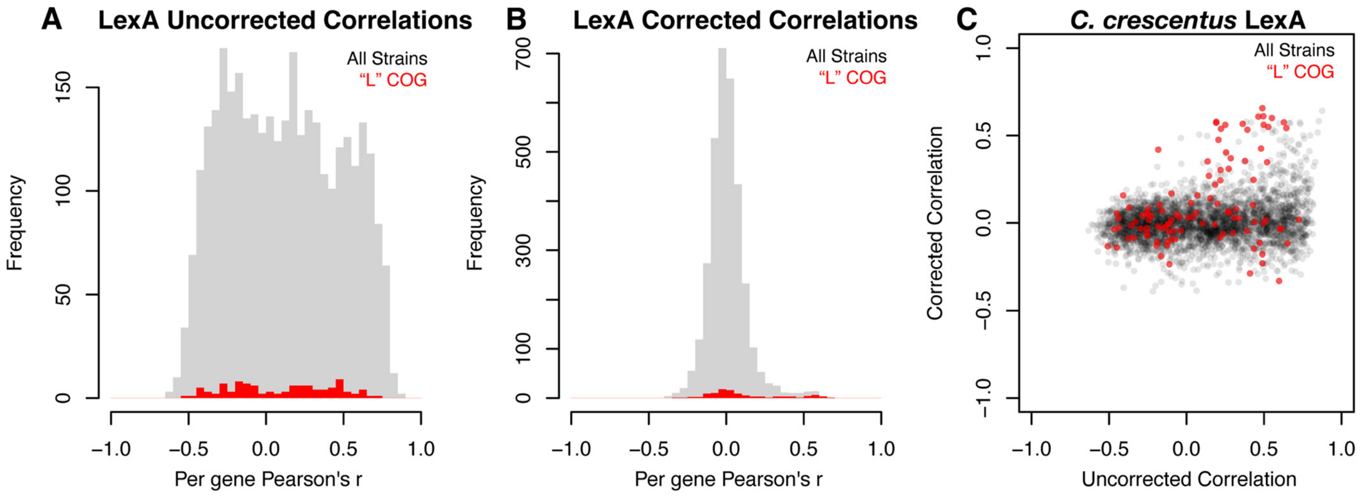

Figure EV3. Correction improves the biological significance of strain-strain correlation with *C. crescentus* LexA (CCNA_01979).

(**A**, **B**) Heatmap of phenotypic correlations between the *C. crescentus lexA* mutant and all other strains in the uncorrected (**A**) and corrected (**B**) datasets. Correlations depicted in red are to genes in the "L - DNA repair and recombination" COG category. (**C**) The same data depicted as an XY-plot, showing that "L - DNA repair and recombination" COG category genes are enriched in the strong correlations of the corrected (but not uncorrected) dataset.

 © The Author(s)

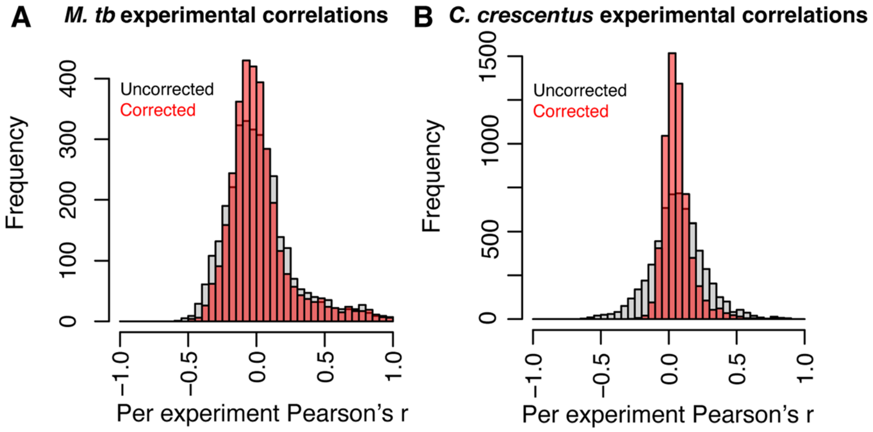

Figure EV4. Correction removes spurious positive and negative correlations between conditions.

(**A**) Histogram of correlations between 81 conditions corrected (red) and uncorrected (gray) for library doubling biases in the *M. tuberculosis* CRISPRi dataset. This dataset contains a higher proportion of same-drug experiments with high correlations to each other, and these high correlations are preserved by the correction. (**B**) Histogram of correlations between 198 conditions corrected (red) and uncorrected (gray) for library doubling biases in the *C. crescentus* Tn-seq dataset.

© The Author(s)

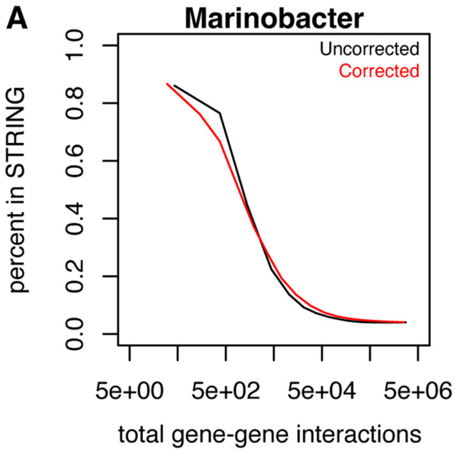

Figure EV5. Curves showing how the fraction of gene–gene interactions in STRING and the number of gene pairs meeting that threshold varies as the correlation threshold is changed.

The data reveals that the correction generally improves recall of known interactions from the STRING database for diverse organisms. (**A**) *Marinobacter adhaerens HP15* (a gamma-proteobacteria). (**B**) *Dechlorosoma suillum PS* (a beta-proteobacteria). (**C**) *C. crescentus* (an alpha-proteobacteria).

 © The Author(s)
